# Multi-faceted nutritional science demonstrated through the prism of sugar: a scoping review on sugar intake and association with quality of life in children and adolescents

**DOI:** 10.1007/s00394-025-03648-3

**Published:** 2025-03-24

**Authors:** Stefania Noerman, Ute Nöthlings, Danijela Ristić-Medić, Bryndís Eva Birgisdóttir, Inge Tetens, Marjukka Kolehmainen, Working Group 1 of the Presidential Activity of Federation of European Nutrition Societies (FENS)

**Affiliations:** 1 Food and Nutrition Science Division, Department of Life Sciences, Chalmers University of Technology, Gothenburg, Sweden; 2 Institute of Nutrition and Food Science, Nutritional Epidemiology, Rheinische-Friedrich-Wilhelms University Bonn, Bonn, Germany; 3 Group for Nutritional Biochemistry and Dietology, Centre of Research Excellence in Nutrition and Metabolism, Institute for Medical Research, National Institute of Republic Serbia, University of Belgrade, Belgrade, Serbia; 4 Unit for Nutrition Research, Faculty of Food Science and Nutrition, University of Iceland and Landspitali University Hospital, Reykjavík, Iceland; 5 Department of Nutrition, Exercise, and Sports, University of Copenhagen, Copenhagen, Denmark; 6 Institute of Public Health and Clinical Nutrition, University of Eastern Finland, Kuopio, Finland

**Keywords:** Nutrition, Methodology, Sugars, Quality of life, Well-being, Children and adolescents

## Abstract

**Background:**

To strengthen the scientific evidence linking dietary sugar consumption with health outcomes, it's essential to look beyond the usual focus on non-communicable diseases (NCDs) and tooth decay. We thus hypothesize that considering other dimensions of health beyond physical health will meaningfully complement the evidence and expand our understanding of the relationship between nutrition and health.

**Purpose:**

The aim of this scoping review was to explore the scientific evidence of an association between dietary sugar intake and quality of life (QoL) among healthy children and adolescents.

**Methods:**

We performed a literature search in three databases (i.e., PubMed, Scopus, and Web of Science). The search included all types of studies assessing dietary sugar intake in association with QoL, in children and adolescents published in English between January 2001 and January 2023.

**Results:**

Twenty-one full-text eligible papers were included in this review: one intervention, two prospective cohort studies, and 18 cross-sectional studies. The number of participants ranged from 25 to 65,000 and age of the participants ranged from 4 to 19 years. The studies differed considerably in exposure and outcome measures. For further qualitative analyses, the studies were categorized into studies related to different dimensions of QoL: food security (n = 4), sleep-related outcomes (n = 5), oral (n = 3) and (mental) health-related QoL (n = 9). Altogether, one study reported a null while the remaining 20 studies found adverse associations between intake of sugar, sugar-sweetened beverages (SSB), or sweets and outcome measures of QoL.

**Conclusion:**

Through this scoping review, a number of scientific studies have revealed an association between sugar intake among children and adolescents and health-related QoL. The findings underscore a negative association. Our review emphasizes the crucial imperative of embracing a broader spectrum of health dimensions to gain a more wholistic understanding of nutrition, especially in collecting science-based evidence for the development of health policies, including dietary guidelines.

## Introduction

The Federation of European Nutrition Societies (FENS) has pointed out a need to identify and develop methods and methodologies that are adapted to the complexity of current nutrition challenges [1] due to the multiple roles that each nutrition component or a nutrient constituent can have as an ingredient, as a food, and as a contributor to a dietary pattern. Inclusion of other aspects of eating, including ethnicity, cultural, economic, and social dimensions, may even further increase the complexity.

During its development throughout the last century, nutrition science has evolved mainly through adapting the medical science-based concepts and methodologies with a reductionist approach. Due to the high prevalence of obesity and non-communicable diseases (NCDs), current nutrition communities put most emphasis on metabolic health. With this perspective, health is seen as the absence of malnutrition and NCDs [2], although the term “health” covers a broader meaning. A meaningful way forward could be to broaden this view from merely the absence of disease or infirmity to the definition of health established by the World Health Organization (WHO) in 1946, as a state of complete physical, mental and social well-being [3]. This definition was even challenged to move the emphasis from a “state” to “the ability to adapt and self-manage in the face of social, physical, and emotional challenges” [4].

Within the same period, emerging new technologies have enabled more precise measurement of biological events. Along with this progress, less defined exposures and “softer” endpoints, such as quality of life (QoL), are now better established, validated, and understood. The WHO defined QoL as “an individual's perception of their position in life in the context of the culture and value systems in which they live and in relation to their goals, expectations, standards and concerns” [5]. With this definition, Qol was suggested to contain three elements: objective living conditions, subjective perceptions of well-being (including evaluations of physical, material, social and emotional health), and personal values and aspirations, which dynamically interact with each other to affect people’s QoL [6]. Therefore, QoL encompasses individual’s overall well-being and satisfaction with various aspects of life, including physical, mental, emotional, and social domains.

Although a reductionist approach is necessary to provide scientific understanding on the molecular mechanisms on how certain components affect health or risk of diseases [7], it appears insufficient for understanding the role of nutrition and surrounding nutrition-related behaviours in relation to individual’s overall health and well-being. The reductionistic approach hence needs to be considered as only one layer complementary to a more wholistic approach [1, 8, 9]. A wholistic approach will require inclusion of other dimensions [7] into a conceptual framework to fully embrace the complexities and multi-dimensional aspects of nutrition science in all its nuances and to allow the scientific evidence to be translatable into policies and practices. Such a framework, however, needs to be first conceptualized and validated in nutrition sciences settings.

The ‘sugar debate’ has a long history in modern nutrition science [2], illustrating that a science-based approach is not always straight-forward. Current sugar reference values have been built on reductionist approaches with slightly different strategies and translations into policy, for example, by WHO [10], the UK (the EatWell Guide) [11], the Nordic Nutrition Recommendations [12], the Dietary Guidelines for Americans [13], etc*.* Dietary guidelines commonly recommend low intake of added sugar or of sugar-rich foods and beverages because of its association with obesity and metabolic diseases, as well as dental problems [14–16]. In a recent scientific opinion on a tolerable upper intake level for dietary sugars, the European Food Safety Authority (EFSA) identified 120 eligible studies linking intake of sugars to risk of adverse health effects such as chronic metabolic diseases, pregnancy-related effects, and dental caries using a risk assessment approach with systematic reviews and meta-analyses [17]*.* The surrounding factors motivating dietary choices or other endpoints related to the broader definition of health, unfortunately, have not been considered.

### Objective

With the complex role of sugar in social settings and cultural context, we hence set out to add another layer in the conceptual framework towards a more holistic understanding of the relationship between "sugar and health". The aim of this scoping review was to explore the scientific evidence of an association between dietary sugar intake and quality of life (QoL) among healthy children and adolescents. We focused on the age group of children and adolescents because of its relevance to develop dietary habits and taste preferences, and because it is often underrepresented in nutrition studies [18–20]. We hypothesized a positive association between sugar intake and QoL in children and adolescents, which may differ from its association with metabolic and oral health.

## Methods

### Our approach

We used a scoping review approach to explore a complex issue that has not been reviewed comprehensively in the past and to highlight the knowledge gap in the field.

### Development of a framework

A framework for sugar intake in relation to a wider range of health-related outcomes emerged based on the internal discussion among the authors as well as with the other members of FENS Working Group One as part of the FENS presidential activity that aimed to improve standards in nutrition science by evaluating the current concepts and methodologies [1]. Sugar was defined as an ingredient, as part of a sugar-rich food or beverage and as a contributor to a dietary pattern. “Health” was defined broadly as maintenance of health/absence of diseases, including NCDs and dental caries. This definition also includes QoL, understood as an individual’s overall well-being and satisfaction with various aspects of life, including physical, mental and social aspects [3]. A recent proposal suggested health as a dynamic response to physical and psychological exposures, including the social aspects [21]. While having children and adolescents as the target groups, it was acknowledged that QoL cannot be separated from other values and confounding factors in the families, including a potential interaction with socioeconomic status (SES).

### Literature search

Using a PICOTSS framework: Population, Intervention (or exposure), Comparator(s), Outcome(s), Timing, Setting(s), Study design.

*Target population*: Healthy children and adolescents 2–18 years of age.

*Exposure*: Sugars are defined as the different subgroups of sugars: sugar naturally present in whole food, free sugars, and foods or beverages rich in added sugars including sweets, cakes, and sugar-sweetened beverages (SSB).

*Comparators*: Studies with comparable controls.

*Outcome variable*: QoL and well-being. Various definitions of QoL and well-being have been previously published [22, 23]. Different studies provide their classifications of child well-being domains, but many of them coincide to focus on cognitive, physical, psychological, educational, social, or behavioural domains to define and measure the feeling and perception of wellness in children and adolescents, either self-reported or reported by the parents.

*Timings*: Cross-sectional, prospective studies, and follow-up of intervention trials.

*Settings*: All settings where healthy children and adolescents spend time.

*Study design*: Observational including cross-sectional and prospective studies and intervention studies.

The search was performed in PubMed, Scopus, Web of Science for articles published in English using the following search terms: (sugar* OR sucrose OR sweet* OR cake* OR comforting food* OR comfort) AND (intake OR meal OR ingestion OR consumption) AND health* AND (famil* OR child* OR infant* OR adolescent* OR teenage* OR youth* OR kindergarten OR daycare OR school* OR student* OR pupil*) AND ((wellbeing OR "well-being" OR "well being" OR quality of life OR life quality) NOT (oral OR teeth OR caries OR diabetes OR coronary OR heart OR cardiac OR cardio OR fatty liver)).

The literature search was performed in two rounds. The first was performed on October 13th, 2021 for articles published on January 1st, 2001–October 13th, 2021, and the second to cover publications in the additional months up until January 23rd, 2023.

### Organizing the results by use of the framework

Extraction of the articles, removal of duplicates, and screening of the titles and abstracts were performed by one person. Full-text screening and extraction of the information were performed by splitting the task among the remaining team members in duplicates. Conflicts were resolved with an additional discussion during meetings with the whole group if necessary. We summarized the findings from full-text eligible papers to tables based on the main categories of outcome measures.

## Results

### The sugar framework

**Fig. 1:**
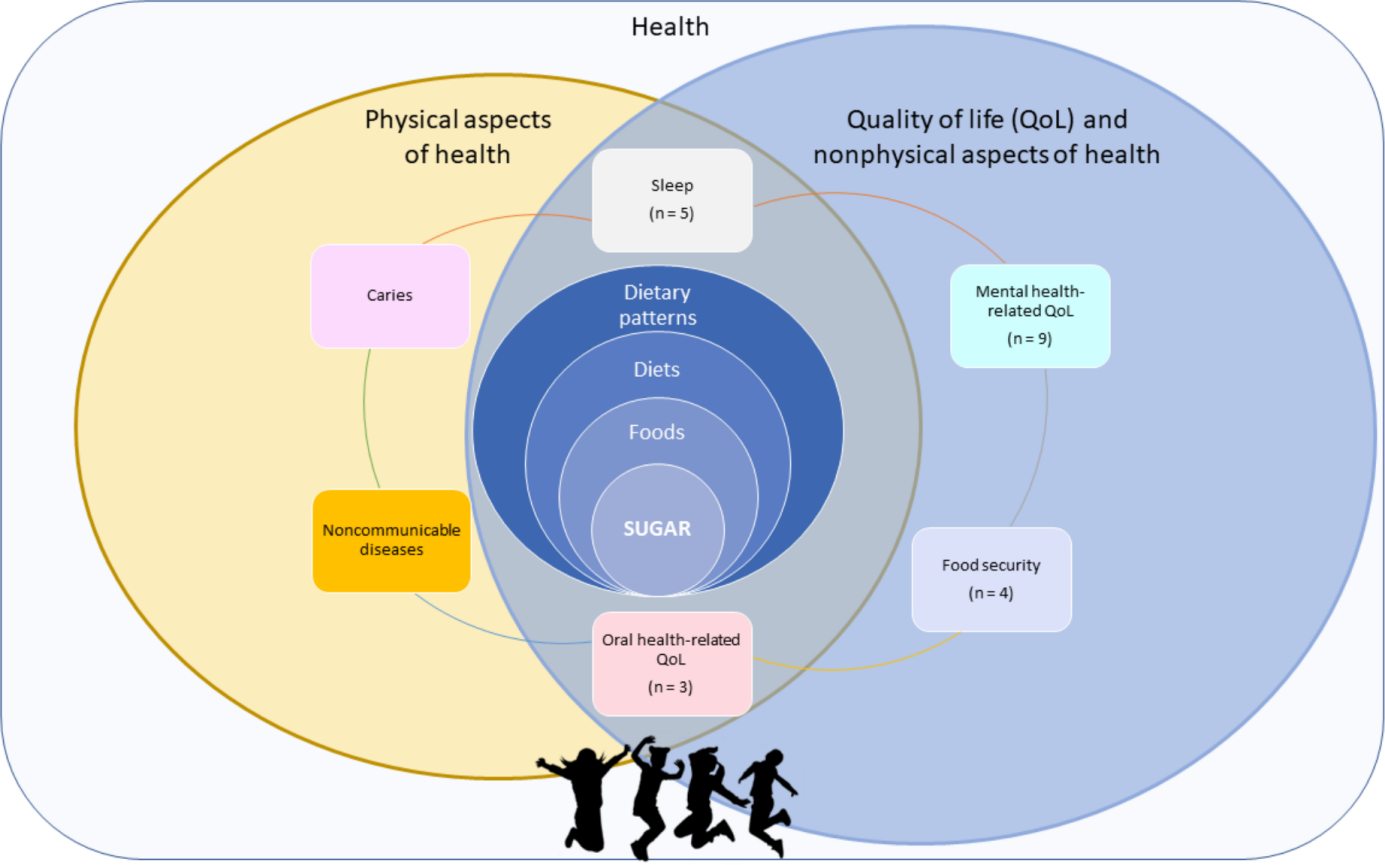
The framework developed within this scoping review for exploring the relationship between intake of sugar and health-related outcomes in children and adolescents

The framework is presented in Fig. 1. We focused on the right side of the framework about the nonphysical aspect of health.

### Literature search

**Fig. 2:**
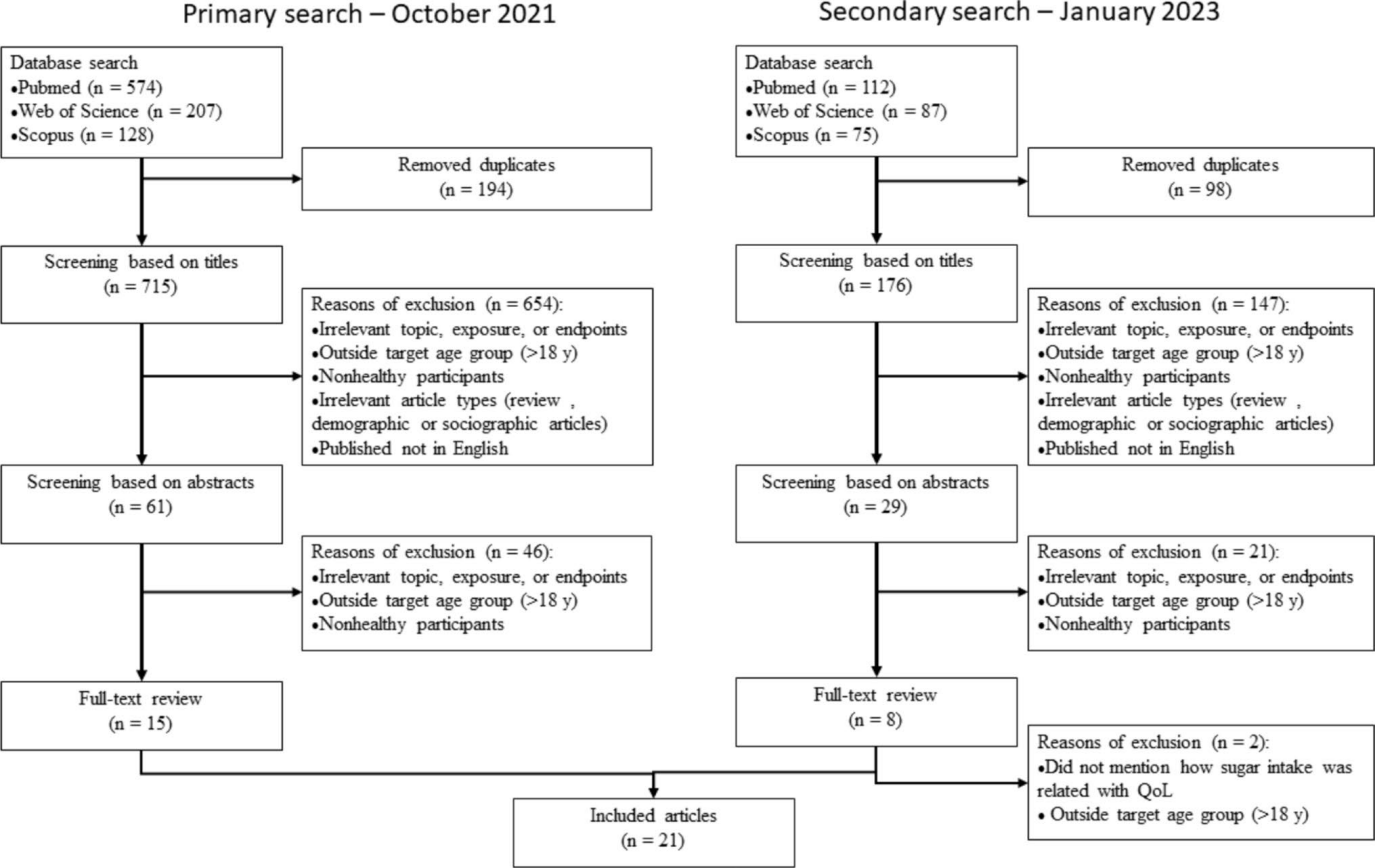
PRISMA flowchart

The search yielded 715 and 176 titles after removal of duplicates from the first and second search, respectively. After screening of titles, 61 and 29 papers were included for abstract screening. After screening the abstracts, 15 and 8 articles were included in the full-text review. Eventually, 21 articles were included in this scoping review (Fig. 2).

We mapped the associations of the consumption of sugar, sugar-sweetened beverages, or respective sugar-containing food from the included 21 studies with various outcomes of QoL. Upon review of the individual studies, the outcomes were grouped into four categories within QoL (Fig. 1), i.e., food security (Table 1), sleep (Table 2), health-related QoL (Table 3), and QoL related to oral health (Table 4). Among these 21 studies, ten were conducted among children [24–29, 31, 38, 39, 43] and eleven among adolescents [30, 32, 33, 35–37, 40–42, 44].

**Table 1:** Overview of studies on the association between consumption of sugar and sugar-sweetened beverages (SSBs) in the diets of children/adolescents and food insecurity

Author (year)	Country	Study type	Study population^a^	Exposure	Dietary assessment methods	Outcome (QoL indicator)	QoL assessment method	Main findings	References
Lee (2019)	USA	Cross-sectional	n = 218 8–12 years	SSB	24 h dietary recall	Food insecurity	Parent-reported US Household Food Security Survey	Children with food insecurity had higher SSB consumption (0.73 (0.53–0.93) compared to 0.43 (0.31–0.55) 8-fl oz; *p* 0.011), lower intake of total energy (1378 (1215–1541) compared to 1704 (1607–1801) kcal; *p* < 0.001) and whole fruits (0.16 (0.05–0.27) compared to 0.29 (0.23–0.36) cup-equivalent; *p* 0.044). The association was significant on weekend day but not on weekday	[24]
Landry (2019)	USA	Cross-sectional	n = 598 9.2 years (7–13) 45% male	HEI including added sugar	24 h dietary recall	Food insecurity	Child Food Security Assessment (CFSA)	Children with food insecurity had higher HEI-component score of added sugar (7.4 vs. 8.0, *p* = 0.002) and lower dietary quality (indicated by HEI-2015; (β = − 3.17; 95% CI = − 5.28, − 1.06; *p* = 0.003)	[25]
Tan (2019)	USA	Cross-sectional	n = 3547 9–12 years	Dietary pattern (HEI-2010)^b^ including sugar and total energy intakes	24 h dietary recalls	Food insecurity	Self-reported 5-item Child Food Security Assessment (CFSA)	Girls with food insecurity had higher intake of energy, total and saturated fat, carbohydrates, and energy from snacks. No statistically significant differences were found in boys. No statistically difference consumption in SSB intake either in either sex (boys β = − 10.7; 95% CI − 24.6, 3.3 kcal; girls β = 5.3; 95% CI − 15.5, 26.1 kcal) in the highest quartile of CFSA score	[26]
Fram (2015)	USA	Cross-sectional	n = 3605 9–11 years 49.7% male	HEI 2005 including added sugar	Diary-assisted 24 h-dietary recall (on school day, not weekend)	Child food insecurity	Child food insecurity (CFI), health behaviours (diet, physical activity)	Greater levels of CFI were associated with higher consumption of sugar (β = 0.79; 95% CI 0.0098, 1.57; p 0.05) g/day, energy (β = 49.61; 95% CI 9.80, 89.43; *p* 0.02) kJ/day. A child at the highest level of CFI, on average, consumed 8 g/day of sugar more than a food-secure child	[27]

^a^n; mean age (range) years; sex (% male)

^b^*HEI* Healthy Eating Index, *SSB* sugar-sweetened beverages

**Table 2:** Overview of studies on the association between sugar and sugar-sweetened beverage (SSB) intake in the diets of children/adolescents and sleep-related endpoints (bruxism, different dimensions, duration, disturbances)

Author (year)	Country	Study type	Study population^a^	Exposure	Dietary assessment methods	Outcome (QoL indicator)	QoL assessment method	Main findings	References
Restrepo (2021)	Colombia	Cross-sectional	n = 440 6.2 years (4–8) 51% male	Food and beverages containing added sugar; frequency of intake per week	FFQ (Health Behaviour in School-Aged Children Food-Frequency Questionnaire)	Sleep bruxism	Children's Sleep Habits Questionnaire (CSHQ)	Positive correlation of possible sleep bruxism with added sugar-consumption (Rho = 0.7; *p* = 0.03)	[28]
Komrij (2021)	Netherlands	Prospective	n = 1180 6.6 years (4–13) 48% male	SSB intake measured at the beginning and at the end of 3 years	FFQ-type	Change in sleep duration over a 3-year period	Parent-reported sleep window	No association between changes in sleep duration and changes in SSB consumption (estimate 0.00; 95% CI − 0.01 to 0.02; *p* = 0.69)	[29]
Khan (2020)	Bangladesh	Cross-sectional	n = 2742 12–15 years 63% male	SSB (exclusion of diet soft drinks)	FFQ-type (30 days)	Anxiety induced sleep disturbance	12-months anxiety induced sleep disturbance	Positive association of carbonated soft drinks consumption > 3×/day with increased sleep disturbances (OR 2.05 (95% CI 1.01–4.18)	[30]
Morrissey (2019)	Australia	Cross-sectional	n = 2253 8.8–13.5 years 50% males	SSB and snack foods	FFQ-type ("Simple dietary questionnaire")	Sleep dimensions	16-item questionnaire developed from previous studies	Positive association with SSB (twice or more a day and often/almost always 1 h before bed) and three or more sleep dimensions scored as poor (*p* = 0.003 and *p* > 0.001, respectively). Positive associations of snack consumption (twice or more a day) with three or more sleep dimensions scored as poor (*p* = 0.003)	[31]
Gan (2019)	Malaysia	Cross-sectional	n = 421 13.3 years (12–16) 42% males	SSB	FFQ-type (7 days)	Sleep quality	19-item Pittsburgh Sleep Quality Index (PSQI)	Positive association between poorer sleep quality and higher consumption of SSB (ß = 0.228, *p* < 0.001)	[32]

*FFQ* food-frequency questionnaire, *HEI* Healthy Eating Index, *ND* not defined, *OR* odds ratio, *SSB* sugar-sweetened beverages, *PA* physical activity, *QoL* quality of life

^a^n; mean age (range) years; sex (% male)

**Table 3:** Overview of studies on association between sugar and sugar-sweetened beverage (SSB) intake in the diets of children/adolescents and Health-Related Quality of Life (HRQoL), life satisfaction or well-being

Author (year)	Country	Study type	Study population^a^	Exposure	Dietary assessment methods	Outcome (QoL indicator)	QoL assessment method	Main findings	References
Qin (2021)	China	Cross-sectional	n = 4388 13.9 years (9–17) 50.2% male	SSB	FFQ (7 days)	HRQoL (utility score)	Child Health Utility 9D (CHU9D) instrument	Inverse association of consumption of SSB (mean difference of weekly consumption frequency = − 0.02; 95% CI = − 0.03, − 0.01) with HRQoL scores	[33]
Walsh (2020)	Europe, North America	Cross-sectional	n = 32,884 15 years (14.6–16.5) 48.3% male	SSB and sweets	FFQ-type (7 days)	Mental well-being indices (low life satisfaction, psychosomatic complaints)	Cantril Ladder (life satisfaction), HBSC Symptom checklist (psychosomatic complaints)	Positive associations of SSB consumption and psychosomatic complaints (r = 0.060, *p* < 0.01), n.s. inverse association with low life satisfaction	[34]
Falbe (2019)	North America	Inter-vention	n = 25^b^ 13–18 years 28% male	SSB withdrawal (3-day SSB cessation)	n.a	Withdrawal symptoms, overall well-being	Modified scale of mood, behaviour, and physical symptoms of caffeine withdrawal and dimensions of mood, including SSB craving	During cessation, participants reported headache (mean ± SD + 0.4 (+ 86%) ± 0.8) and decreased feelings of motivation 0.6 (− 25%) ± 0.9 and ability to concentrate (− 0.4 (− 15%) ± 1.0), showing potentially addictive behaviour	[35]
Hong (2017)	Korea	Cross-sectional	n = 65,212 15.1 years 52.2% male	Soft, highly caffeinated and sweetened drinks (SSB)	FFQ (7 days)	Psychological well-being and distress	5-item questionnaires for each of the variables, except for the depression symptoms (binary)	Negative associations of intake of SSB with self-reported health (adjusted odds ratio = 0.58 (95% CI 0.58–0.70), happiness (0.68 (0.57–0.82), sleep satisfaction (0.82 (0.66–1.01), and positive association with perceived stress (2.08 (1.73–2.50)) and depression symptoms (1.97 (1.67–2.32) in participants with the highest intake frequency (3 + times/day)	[36]
Kohl-boeck (2012)	Germany	Cross-sectional	n = 3361 11.15 years (10–13) 51% male	High sugar foods	FFQ	Behavioural problems	Parent-reported Strengths and Difficulties Questionnaire (SDQ) (emotional symptoms, conduct problems and hyperactivity/inattention)	Positive association of increased intake of confectionery with odds of having emotional symptoms (OR 1.19, 95% CI 1.08–1.32] compared to children with low intake	[37]
Gete (2022)	Australia	Cross-sectional	n = 1448 pairs of mothers and children age 5–12 years	HEI	Mothers: a semi-quantitative and validated 101-item FFQ in the past 12 months children: a semi-quantitative and validated 28-item Children Dietary Questionnaire in the past 7d or 24 h	Childhood behavioural disorders	Strengths and Difficulties Questionnaire	20% of the total effect of the poor adherence to pre-pregnancy diet quality on the risk of offspring behavioural problems was mediated by childhood high consumptions of fats and sugar (OR mediated effect 1.10; 96% CI 1.03, 1.17; total effect OR 1.83; 96% CI 1.20, 2.78)	[38]
Juton (2022)	Spain	Prospective, 15-month follow-up	n = 1371 children (50% male) age = 10.1 ± 0.6 years	Adherence to Mediterranean diet	16-item KidMed	HRQoL	KIDSCREEN-10 index questionnaire	Consumption of sweets and candies was negatively associated with HRQoL at follow-up (OR 0.65; 95% CI 0.46, 0.91)	[39]
Geraets (2022)	Luxembourg	Cross-sectional	n = 7529 51.7% girls age 14.9 ± 2.1 years	Frequency intake of fruits, vegetables, sweets, and sugar-containing drinks	FFQ	Self-reported health, multiple health complaints, life-satisfaction, health behaviours	Excellent health question: "what would you say your health is?"; multiple health complaints: Health Behavior in School-aged Children (HBSC)-Symptom Checklist; Life-satisfaction: Cantril Ladder^c^	A group of individuals marked with high sugar consumption reported lower score in self-reported health parameters (OR 0.79; 95% CI 0.68, 0.92), lower life-satisfaction (O 0.74; 95% CI 0.58, 0.95), and more multiple health complaints (OR 1.34; 95% CI 1.14, 1.56)	[40]
Ra (2022)	South Korea	Cross-sectional	n = 24,006 high-school students, unknown age and gender proportion	SSB and fast-food consumption	2-item FFQ for SSB and a single-item FFQ for fast-food consumption in the past 7 days	Stress, depressive symptoms, suicidal ideation	Stress: single-item questionnaire depressive symptoms: single-item questionnaire about participants sadness and hopelessness in the past 12 months suicidal ideation: a single-item questionnaire whether participants have seriously considered committing suicide in the past 12 months	SSB consumption was associated with higher stress (OR 1.20; 95% CI 1.11, 1.29), depressive symptoms (OR 1.19; 95% CI 1.09, 1.30), and suicide ideation (1.18; 95% CI 1.05, 1.32) in the highest tertile	[41]

^a^n; mean age (range) years; sex (% male)

^b^overweight/obese individuals who typically consume 3 or more servings of SSB per day

^c^Explanation on each tool can be found as supplemental information of the paper [40]

**Table 4:** Overview of studies on association between consumption of cariogenic foods/diet in children/adolescents and Quality of Life related to oral health (OHRQoL)

Author (year)	Country	Study type	Study population^a^	Exposure^b^	Dietary assessment methods	Outcome (QoL indicator)	QoL assessment method	Main findings	References
Montero (2018)	Portugal	Cross-sectional	n = 782 11–17 years 44.5% male	Cariostatic food versus cariogenic food	FFQ	Oral-health-related quality of life (OHRQoL)^c^	49-items Oral Health Impact Profile questionnaire (OHIP-49)	Adolescents consuming sweet-containing foods or beverages > 1×/week reported lower OHRQoL, indicated by positive associations with e.g., physical, mental, and social disability components as well as psychological discomfort	[42]
Freire (2022)	Portugal	Cross-sectional	n = 1475 age 4.1 ± 0.8 years 51.3% male	Frequent intake of cariogenic foods (daily or most of the days)	Specially developed questionnaire related to oral health	Oral-health-related quality of life (OHRQoL)	Portuguese version of Early Childhood Oral Health Impact Scale (ECOHIS)	Children with frequent intake of cariogenic foods and drinks had a lower OHRQoL (mean (SD) = 1.8 (3.5), *p* < 0.001)	[43]
Nora (2022)	Brazil	Cross-sectional	n = 1197 age 15–19 years 54.6% male	Cariogenic diet	Self-perception of a healthy diet and the frequency of consumption of sugary foods and drinks	Oral-health-related quality of life (OHRQoL)	Oral Health Impact Profile-14	A cariogenic diet was associated with poorer OHRQoL (standardized coefficient 0.16, *p* < 0.01)	[44]

^a^n; mean age (range) years; sex (% male)

^b^In Montero et al. [42], the participants filled in FFQ with various food items categorized to cariostatic and cariogenic food groups according to the classification of American Dietetic Association [45] and Palmer [46]. In Freire [43], participants filled in a questionnaire with a question on “frequent intake of cariogenic food/drinks (daily or most of the days)”. In Nora [44], the question was about daily consumption of sugary food and drinks

^c^Higher score indicates lower OHRQoL

Table 1 shows four cross-sectional studies investigating the association between sugar and SSB consumption and food insecurity in children [24–27]. Noteworthy, all studies were based in the North America. Though they may include mixed races, (two studies consisted of mostly Hispanic, one mostly Caucasian, one did not report), it may still restrict the generalizability of the findings to other study populations.

A total of five studies [28–32] addressed a wide range of sleep-related outcomes (Table 2), covering sleep duration, sleep disturbances, sleep bruxism, or other sleep related outcomes, such as quality of sleep. One of these studies was a prospective study [29] and the other studies were cross-sectional [28, 30–32]. Most study populations included adolescents, with one addressing children [28]. The studies were also quite well distributed geographically, with representations from Asia (n = 2) [30, 32], Europe (n = 1) [29], Australia (n = 1) [31], and Latin America (n = 1) [28]. Intake of sugar was analysed at the level of foods, with none of the studies referring specifically to/estimating the actual amount of sugar intake. Four of five studies examined SSB, except one study which included sugar-rich foods and beverages. Most of the studies used short FFQ-type questionnaires focusing on the food groups of interest rather than validated dietary assessment methods. With respect to the measurement of sleep with all its dimensions, each study used a different method, and two of the methods were reported to have been standardized [28, 32]. All four cross-sectional studies reported positive associations between intake of SSB/foods with added sugar and poor sleep quality (reduced sleep duration and quality, higher sleep bruxism and more sleep problems), whereas the one prospective study [29] looking into change in sleep duration over a 3-year period found no associations.

Table 3 showed some studies addressing several dimensions of nonphysical health and health-related QoL: mental/psychological and overall wellbeing, behavioural problems, and health-related QoL. A total of nine studies [33–35, 37–41, 47], mainly cross-sectional with one intervention [35] and one prospective study [39] were found. The studies were conducted in Asia (n = 3) [33, 36, 41], Europe (n = 4) [34, 37, 39, 40], Australia (n = 1) [38], and North America (n = 2) [34, 35]. The definitions and assessments of outcomes varied considerably across the studies, ranging from health-related QoL indices, life satisfaction, psychosomatic complaints, mood, behaviour, emotional and physical symptoms, stress, depression, to suicidal ideation. Also, the exposures were different, ranking from SSB, sweets, to sugar as a component that is discouraged in Healthy Eating Index (HEI) or Mediterranean diet. All studies found associations between higher intake of sugar/sugary foods and drinks with lower health-related QoL measures, indicated by lower life satisfaction and happiness as well as higher emotional problems, psychosomatic and multiple health complaints, perceived stress, depression symptoms, and suicide ideation.

Table 4 showed the association between cariogenic foods or diet and oral health-related QoL (OHRQoL), measured using oral health impact profile questionnaire (physical, mental, and social disability) for two studies conducted among adolescents [42, 44] and early childhood oral health impact scale for one study in children [43]. Two studies were conducted in Europe [42, 43] and one in Latin America [44]. All studies reported associations between consumption of cariogenic food and drinks with lower OHRQoL.

## Discussion

We explored the relationship between sugar and quality of life in children and adolescents as an attempt to challenge the conventional realms of a relationship between sugar and health. In contrast to our initial hypothesis, we found consistent adverse associations across most of the studies between sugar intake and measures of QoL, including sleep, food security, and health-related QoL, despite the limited number of papers and the high variability in the sugar exposure and outcome measures. Besides giving further support to the inverse association between sugar intake and health-related outcomes underlying the current dietary reference values and Food Based Dietary Guidelines [10–12, 17], this finding may underline the importance of embracing the multidimensional aspects of health, taking into account of the broad range of exposures and outcome measures or measurement tools.

Common behaviors of consuming sugar-rich foods containing added sugar were presumed to be associated with the context of social gathering, relaxation, and comfort, which may be tightly associated with QoL. Sweet and fatty foods have previously been associated with feelings of pleasure in adult populations [48]. High SSB consumption, in contrast, was shown to be driven by multiple factors, from the children’s preference and behaviours (e.g. screen time, snack consumption), parental factors (e.g., lower SES, lower age, SSB consumption, using food as reward, etc.) [49], which may differ from factors driving sugary food consumption. This study hence highlights the multidimensionality of both sugar intake and QoL. Embracing the full complexity of both sugar intake and QoL taking into account all different aspects surrounding this relationship is hence necessary, despite challenges in merging the highly variable exposure and outcome measures, which interestingly, seemed consistent in this study.

Since most of the included studies were cross-sectional, it is important to remember the possibility of reverse causation, i.e., higher sugar intake in those with lower QoL, especially in case of food insecurity. However, the consistent associations in almost all the included studies may imply underlying causal relationships, which deserves further investigations. Embracing the full complexity of QoL taking into account all its different aspects is necessary, despite challenges in establishing the causal relationship and merging highly variable outcomes.

The populations in the studies that were included in the present scoping review represented children between 4 and 8 years of age and adolescents aged 12–19. Intake of sugar-sweetened foods and beverages is of concern to children and adolescents, especially due to the low nutritional value of these foods. These foods contain energy and replace the more nutritious foods in children’s and adolescent’s diets [50]. Eating behavior and food choices are highly complex processes driven by physiological (i.e. genotypes, phenotypes, metabolic functions, homeostatic balance), psychological (i.e. mood, hedonic), as well as surrounding socioeconomic factors [51]. These aspects affect not only how much, but also when and on which occasions people eat. Many choices affecting sugar intake are shaped by interpersonal relationships and social activities, which may vary from one culture to another. Similarly, sugar may be one of the most affordable sources of energy, which may encourage its preferred intake over other dietary components with higher nutritional quality. As the juvenile/adolescent period has been shown to be an important period for the development of food preferences and habits with long-lasting health consequences [18–20], the high intake of sugar-rich foods and beverages becomes a concern if it is retained throughout life.

The adolescent period is an important period which may determine the onset of anxiety later in life [52]. Consumption of hypercaloric diet has been proposed to influence the neuroendocrine stress systems and the maturation of neural circuitry supporting emotion regulation [52]. Additionally, children and adolescent may be exposed to marketing of sugar-rich foods that may contribute to health-detrimental eating behaviors [53]. In a meta-analysis of randomized trials it was shown that children exposed to advertisement of energy-dense sugary products were more likely to consume those foods and increase their dietary intake, especially in the younger age group (≤ 8 years of age) [54]. The fast foods and sugar-rich foods and beverages may also be consumed in connection to stressed situation or along with emotional eating in adolescent population [55, 56]. The opposite direction seemed to also apply, as the consumption of imprudent diets rich in sugar-rich beverages, processed foods, and foods rich in saturated fatty acids was linked to an increased risk of depression in adolescents [57, 58].

Similar adverse associations between sugar intake and various measures of QoL have been previously reported in adult populations. Among university students aged 18–25 year old, higher intake of added sugars and SSB was observed in students with food insecurity [59, 60]. In another study conducted at university students, overconsumption of high-fat and high-sugar food was associated with poor sleep quality [61]. Higher consumption of SSB in Spanish adults has also been associated with lower score in the physical component of a validated questionnaire of health-related QoL [62]. Despite limited number of studies and lack of systematic review in this field, this finding hence showed a good concordance between children and adult populations, which may deserve a future in-depth examination on the association between sugar intake and QoL.

Most of the studies in our literature review did not include information about the amount of actual sugar intake because we did not aim to establish the cut-off limit nor expect to observe a linear dose–response relationship between sugar intake and lower QoL. The intake of SSB or other sugar-rich foods was measured either by using FFQ or 24-h foods recall, with no well-validated scales, scores (such as HEI with added sugar) or questionnaires developed for the purpose. One of the possible reasons could be due to challenges in quantifying sugar intake from sugar-rich foods and beverages, especially when the exposure in focus was dietary patterns. Additionally, included studies in this review did not classify added sugar intake as according to the cut-off values often used in official recommendations. In the recently published EFSA scientific opinion on a tolerable upper intake level for dietary sugars, challenges of identifying studies with intake of less than 10% energy intake were mentioned [63]. Thus, we cannot use current study to estimate the actual amount of sugar associated adversely with QoL.

In the research literature, the agreement on how to measure children’s QoL seems lacking. The outcome related to quality of sleep such as sleep bruxism; sleep duration and sleep disturbance may have a strong impact on the QoL. However, studies are not easily comparable with differing sleep-related outcomes and with differing methods used, such as parts of the questionnaires from the previous studies, the observations of the parents, or sleep habits questionnaire or sleep quality index. Other outcome measures were mental well-being, oral health, physiological well-being, or food security. Food insecurity as the outcome was measured differently in the studies. For example, parent reports and child food security assessment tools (CFSA) were used. The difference in methods may interfere with the interpretation and reliability of our findings.

Despite the lack of quantitative measures in both exposures and outcomes, the consistent inverse association between sugar intake and measures of QoL across these studies was striking. Because of the high heterogeneity of sugar exposure and QoL outcome measures, our attempt was to find emerging patterns from included studies and highlight the knowledge gaps in the field instead of estimate the strength of the association between sugar intake and QoL. We were surprised by the number of studies appearing during the secondary literature search, which may imply a growing interest in this field, or emerging interdisciplinary approaches in solving nutrition-related challenges in the past few years. Given the findings of our study, we are convinced that inclusion of multidimensional aspects enabled by collaborations with other fields will capture a wider perspective to better understand underlying relationship between diet and health, which will provide stronger evidence for policymaking, including formulation of nutrient recommendations and dietary guidelines.

There are several lessons learned from this review. First, we observe a gap in the scientific literature for additional understanding of the role of sugar in QoL and well-being. Many previous studies focus on hard endpoints such as risk of cardiometabolic diseases, indirect risk markers, or other quantifiable measures. Nevertheless, health is not merely about being free of disease or being responsive to health disturbance and treatment of symptoms, but the capacity to respond to challenges within the windows of homeostatic balance [21]. Additionally, health comprises not only the physical aspect, but also the emotional, social, and psychological well-being. QoL was picked based on the availability of quantifiable measures using validated questionnaires. However, in this study we observed that QoL also embraces the positive and active aspects in health maintenance and prevention strategy as well as various aspects of life, such as sleep quality, social aspects (mood, behaviour, emotional and depression symptoms), and food security. Some of the measures are well described with validated methods, while some others may not be quantifiable with validated questionnaires or other quantitative measures. This exercise hence shows how current nutrition science practices are skewed towards mostly quantifiable physical aspects of health, which overlook other aspects shaping one’s physical and mental well-being. Given the intricacy involved, reductionist approaches that solely focus on quantitative aspects of medical sciences may fall short in offering a comprehensive understanding of nutrition science.

Our scoping review suggests that inclusion of different methodologies and endpoints measuring both physical and mental health from different angles such as QoL illustrates the value of adding additional layers to the existing as a way towards a more holistic approach. Although no study with qualitative behavioral data was included in this review, inclusion of a more wholistic lens in the future is highly encouraged. Based on our findings from sugar and health, we argue that when considering the complexity, reductionist approaches focusing only on quantitative aspects of medical sciences may not suffice to provide wholistic insight on nutrition sciences. To this end, we hypothesize that by complementing currently practiced reductionist approaches with additional layers inherent in nutrition, we will arrive at a more wholistic approach where important new dimensions of the relationship between nutrition and health may lead the field of nutrition science forward.

Second, the quantitative aspects of nutrition science as derived from medical studies always require exposure/dosage information. This information, however, may not always be applicable as people would have different levels of e.g., energy intake, energy expenditure, and factors behind dietary choices. None of the included studies mentioned the actual amount of sugar intake, but SSB (10 studies), sugar-rich food and beverages (4 studies), or dietary patterns (7 studies), which hinder translation of the current review to draw any dose–response relationship or recommend the maximum amount of intake without possible harmful consequence. Though no advice on sugar intake in children and adolescents related to their QoL can be derived using our approach, we underline some of the challenges that were also depicted by the EFSA’s scientific opinion on the sugar intake [63].

We acknowledge that we ended up with a relatively low number of studies, which may represent the high proportion of studies with reductionist approach or be attributable to traditional medical point-of-view in article screening with restricted inclusion criteria, study population, or outcome measures. Similarly, many of the studies stemmed from other disciplines than nutrition sciences, which may have influenced methodologies. Most (18 of 21 studies) were cross-sectional as only a limited number of other study designs were screened. In those studies, higher sugar intake coexists with other unfavorable health behaviors, such as lower physical activity, higher screen time [31], lower dietary quality [24–26, 31], including high frequency of SSB intake. Therefore, we cannot resolve if the SSB intake was the cause, effect, or an effect modifier of the low-quality diet. Despite moderate to good correlation between frequency of SSB intake and sugar intake [64, 65], it may not suffice to represent total sugar intake due to varying SSB consumption patterns among individuals and populations, varying sugar content in SSBs, and other sources of added sugars in diets than SSBs. Additionally, food insecurity may act as exposure instead of the outcome of the high sugar intake. As most of the studies were cross-sectional, no directionality or causality can be inferred from these studies, although the consistent associations across studies may infer underlying causal relationship among the included variables. Moreover, the long-term impact of sugar consumption cannot be concluded, either. Not all the included studies reported SES of the study participants, although SES may confound the associations between sugar intake and e.g., food insecurity and other aspects of QoL. Also, the measures of exposures and outcomes differed by age group, e.g., filled by parents for smaller age group and by the adolescents themselves. The immense variation in the study populations, designs, exposures, measurement tools, and main outcomes made it challenging to merge the findings and draw a generalizable conclusion, despite strikingly similar adverse association over such high variations. Considering this diversity, inference was then made based on the observed patterns across study.

Based on the studies reviewed, we suggest that a higher consumption of sugar-rich foods and beverages are likely to have detrimental association with QoL, although no causality and time-related relationship can be established and lacking information on the actual amount of consumed sugar. Many of the studies took insights from other disciplines such as behavioural or social sciences, bringing out contexts where sugar-rich foods and beverages may be related with other outcomes, such as food security, health-related quality of life, and sleep quality. In combination with metabolic and physiological outcomes, these aspects may add a value which may also be of interest for consumers and clients in the healthcare system. In the future, multidimensional aspects of nutrition, considering both reductionist and wholistic approaches, e.g., intervention studies with continuous monitoring of QoL components, and inclusion of other interdisciplinary approaches, such as health perception, more subjective health outcomes, taking into account socioeconomic status, cultural values, and other factors in the society, are hence encouraged. An extra caution is required to overcome challenges of merging both quantitative and qualitative measures to reflect health to fully embrace the complexities in nutrition science and support the translation of nutrition science into food- and nutrition policies, to health professionals, and for communication to the public.

## Abbreviations

FFQ: Food frequency questionnaire
NCD: Noncommunicable diseases
SSB: Sugar-sweetened beverages
QoL: Quality of life
WHO: World Health Organization
